# Ecological niche selection shapes the assembly and diversity of microbial communities in *Casuarina equisetifolia* L.

**DOI:** 10.3389/fpls.2022.988485

**Published:** 2022-10-20

**Authors:** Qi Lin, Ying Wang, Miaomiao Li, Zhixia Xu, Lei Li

**Affiliations:** Ministry of Education Key Laboratory for Ecology of Tropical Islands, Hainan Normal University, Haikou, China

**Keywords:** *Casuarina equisetifolia*, microbiome assembly, compartment niche, plant-associated microorganism, Hainan province, soil–plant continuum

## Abstract

The plant microbiome profoundly affects many aspects of host performance; however, the ecological processes by which plant hosts govern microbiome assembly, function, and dispersal remain largely unknown. Here, we investigated the bacterial and fungal communities in multiple compartment niches (bulk soil, rhizosphere soil, root endosphere, phylloplane, and leaf endosphere) of *Casuarina equisetifolia* L. at three developmental stages in Hainan Province, China. We found that microbiome assemblages along the soil–plant continuum were shaped by the compartment niches. Bacterial diversity and richness decreased from the soils to roots to leaves, with the highest network complexity found in the roots and the lowest found in the phylloplane. However, fungal diversity gradually increased from the soils to roots to phyllosphere, whereas fungal richness decreased from the soils to roots but increased from the roots to phyllosphere; the greatest network complexity was found in bulk soils and the lowest was found in the roots. Different biomarker taxa occurred in the different ecological niches. Bacterial and fungal communities exhibited distinct ecological functions; the former played important roles in maintaining plant growth and providing nutrients, whereas the latter predominantly decomposed organic matter. The bacterial community of *C. equisetifolia* mostly originated from bulk soil, whereas the fungal community was mainly derived from rhizosphere soil and air. Leaf endophytes were positively correlated with organic carbon, and root and soil microorganisms were positively correlated with total nitrogen, total phosphorus, and total potassium. Our findings provide empirical evidence for plant–microbiome interactions and contribute to future research on non-crop management and the manipulation of non-crop microbiomes.

## Introduction

Microorganisms are ubiquitous in plant tissues such as roots, stems, leaves, flowers, and fruits (Lindow and Brandl, 2003; Hacquard et al., 2015). Plants and their inhabiting microbiome together constitute a “holobiont,” with the plant microbiome acting as a secondary genome and a link to host fitness (Vandenkoornhuyse et al., 2015). Therefore, comprehending the mechanisms underlying plant microbiome assembly, function, and dispersal is important for our fundamental understanding of the development of microbiome-based strategies required to maximize plant survival and increase their tolerance to low soil fertility, alkalinity, and salinity.

Assembly of the plant microbiome begins shortly after sowing and develops with plant growth under the influence of stochastic (e.g., random dispersal) and deterministic (e.g., selection mediated by biotic and abiotic factors) processes (Bulgarelli et al., 2013; Müller et al., 2016). In addition to vertical transmission (Müller et al., 2016; Abdelfattah et al., 2021), microorganisms from the soil and air can migrate to and colonize different compartments of plants (Bulgarelli et al., 2013; Compant et al., 2021). A study has highlighted the important contribution of plant developmental stage to plant microbiome assembly such as the physiological needs of plants and the composition of plant secretions (Zhang et al., 2018). However, the mechanisms by which the host and environment shape microbiome assemblages and symbiotic patterns across the soil, root endosphere, and phyllosphere remain largely unknown.

Our understanding of the microbiome assembly mechanism in plants is still in its infancy, with several key questions yet to be answered. The root microbiome is also assembled from soil microorganisms, and based on the composition of microbial communities in the root endosphere and rhizosphere, this assembly may be achieved in two steps: (1) recruitment of rhizosphere microorganisms by plants; (2) entry of microorganisms into the plant (Bulgarelli et al., 2013). Furthermore, the phylloplane is an important interface among plants, microorganisms, and the environment, and microorganisms from the surrounding environment can colonize the leaf endosphere (Lindow and Brandl, 2003). Although the aforementioned hypotheses are reasonable, the microbial composition among the bulk soil, rhizosphere, root endosphere, phylloplane, and leaf endosphere, as well as the relationship between the rhizosphere and phyllosphere, remain unclear. In addition, the roles of microorganisms in different ecological niches have yet to be clarified.

In the late 1950s, *Casuarina equisetifolia* L. was first introduced to China in Guangdong Province (Li et al., 2015), where it serves as an important shelter tree species on the southeastern coast of China (Liu et al., 2020). *C. equisetifolia* is characterized by wind speed reduction and sand fixation, as well as salt and alkali tolerance. Moreover, it is of great importance for the restoration of ecological functions and protection against natural disasters in coastal areas, and it contains abundant endophytic microorganisms that perform important functions related to saline–alkali tolerance, low soil fertility tolerance, and allelopathy (Lin et al., 2008). Previous studies have shown that vertical transmission of endophytes in *C. equisetifolia* affects the dispersal of endophytic fungi from seeds to the leaf endosphere and the transmission of endophytic bacteria from seeds to the root endosphere (Lin, unpublished). However, there has been little investigation of the impact of the soil–plant continuum on the plant microbiome, such as epiphytes and endophytes.

Here, we use *C. equisetifolia* as a model system to fill these key knowledge gaps. Specifically, this study has two objectives: (1) evaluation of the microbiome assembly at different developmental stages (young, mature, and aged forests) and in different compartment niches (bulk soil, rhizosphere soil, root endosphere, phylloplane, and leaf endosphere) of *C. equisetifolia*; (2) identification of the potential sources, dominant taxa, and ecological functions of plant microbial communities, as well as the effects of environmental factors on microorganisms. To achieve these objectives, we use high-throughput sequencing technology and chemical analysis to evaluate samples associated with *C. equisetifolia* at different developmental stages. The following hypotheses are proposed: (1) the microbial community migrates from the soil (a reservoir of microorganisms) to plant roots and then to plant leaves, with a gradual decrease in microbial diversity; (2) bacteria and fungi perform distinct functions in different compartment niches.

## Materials and methods

### Sampling site

*C*. *equisetifolia* forests located in the Guilinyang Coastal Development Zone, Haikou city, Hainan Province, China (N20°01’02”, E110°31’20”) were selected as the study site. This site has a tropical marine monsoon climate with mean annual precipitation of 1,500–2,000 mm and mean relative humidity of 85%. The sampling site has a mean annual temperature of up to 23.8°C and a long sunshine duration (Cai et al., 2010).

### Sample collection and treatment

The bulk soil, rhizosphere soil, roots, and leaves were collected in fall 2020 from three forest plots at different developmental stages (young stand: 5-8 years, mature stand: 15-20 years, aged stand: over 30 years). For root and leaf sampling (50 g for each plot), five individual plants were randomly selected from each plot. Rhizosphere soil that was attached to the same roots was collected (the depth approximately 15 cm), and the soil 0–15 cm away from the roots was collected as bulk soil. Five subsamples were thoroughly mixed as a biological sample for each plot. A total of 45 samples (bulk soils, rhizosphere soils attached to root, roots and leaves) were placed in sterile plastic bags and transported to the laboratory in a cool box with ice packs. The samples were then divided into two parts. One part was subjected to the following protocol: a sterile brush was used to remove the rhizosphere soil from the root surface; for collected leaf epiphytes and removed root epiphytes, we referred to previous methods (Bodenhausen et al., 2013), with some modifications. In brief, 10-15 g of leaves and 3-5 g roots (rhizosphere soils were removed by brush carefully) tissues were submerged in 0.1 M Potassium phosphate buffer (pH 8.0) and subjected to sonication at 40 kHz for 1 min. This procedure was repeated 3 times, and then the buffer was filtered through a 0.22 μm-pore filter. For endophytic DNA, we used the same leaves and roots after further sterilization (Ren et al., 2019), with some modifications. Briefly, we further washed the leaves and roots mentioned above with sterile water, and treated with 75% ethanol for 30 s, followed by an immersion in 5% sodium hypochlorite solution for 5 min,and finally washed with sterile water for 3 times. Sterilization was checked by plating the last washing water on Luria-Bertani (LB) used for bacteria and Potato Dextrose Agar (PDA) used for fungi and incubating at 28°C. We used sterile mortars and pestles with liquid nitrogen to ground the treated leaf and root tissues. For the other part, soil (mixture of rhizosphere and bulk soil), root, and leaf samples were selected, with roots and leaves washed by deionized water, and all samples were baked at 105°C for 30 min, then at 65–80°C for 8–12 h until dry. These samples were then ground separately and sieved to 1 mm for further use.

### DNA extraction, microbial rRNA gene amplification, and sequencing

Total genomic DNA was extracted from the collected samples using the FastDNA^®^ Spin Kit for Soil (MP Biomedicals, U.S). ITS1F (CTTGGTCATTTAGAGGAAAGTAA) and ITS2R (GCTGCGTTCTTCATCGATGC) were used for polymerase chain reaction (PCR) amplification of fungal 18S rRNA, with 338F (ACTCCTACGGGAGGCAGCAG) and 806R (GGACTACHVGGGTWTCTAAT) used for PCR amplification of bacterial 16S rRNA. The PCR reaction mixture contained 12.5 μL Premix Taq DNA polymerase (Takara, China), 0.5 μL (200 μM) of each primer and a 10-ng template DNA, followed by PCR-grade water added to a final volume of 25 µL. The PCR amplification cycling conditions were as follows: initial denaturation at 94°C for 2 min, 30 cycles of denaturing at 94°C for 30 s, annealing at 55°C for 30 s and extension at 72°C for 45 s, with a final 10 min elongation at 72°C. Sequencing was performed on an Illumina Miseq PE300 platform (Majorbio, Shanghai, China).

### Determination of sample chemical properties

We refer to previous methods for analyzing the soil chemistry of leaves, roots and soils (Huang, 2019). The organic carbon, total nitrogen, total phosphorus, and total potassium contents were determined by chromic acid oxidation, Kjeldahl determination of nitrogen, Mo–Sb colorimetric method, and flame photometry, respectively.

### Bioinformatics analysis

We truncated the 300 bp reads at any site receiving an average quality score below 20; we discarded truncated reads shorter than 50 bp containing ambiguous characters. We assembled the resultant sequences according to their overlapped sequence with a minimum of 10bp; the maximum mismatch ratio of an overlapping region was 0.2, and we discarded reads that could not be assembled (Magoč and Salzberg, 2011; Chen et al., 2018). Then, we clustered the optimized sequences into operational taxonomic units (OTUs) using UPARSE 7.1 with 97% sequence similarity level and selected the most abundant sequence for each OTU as a representative sequence. (Edgar, 2013). The taxonomy of each OTU representative sequence was analyzed using RDP Classifier (v.2.2) against the 16S rRNA (Silva v.138) and ITS (Unite v.8.0) gene database in QIIME 1 (Caporaso et al., 2010). Overall, we obtained 2, 385,500 bp sequences, with a total base pair number of 989,402,997 bp for bacteria, and 2,313,685 bp sequences, with a total base pair number of 634,976,872 bp for fungi after optimization, quality control, and filtration.

### Data statistical analysis

Principal coordinate analysis (PCoA) (Calderón et al., 2017) and plotting were performed using ADE4 package in R (v.3.3.1). Alpha diversity indices (Rogers et al., 2016) were calculated using Mothur (v.1.30.2), and box plots were generated using BASE package in R (v.3.3.1). Networkx software (Ramayo-Caldas et al., 2016) was used to analyze the co-occurrence network of the distribution (top 50 species in relative abundance, absolute value of Spearman correlation coefficient ≥ 0.5, *P*-value < 0.01). Community bar plot analysis (Ji et al., 2017) was implemented PANDAS package in Python (v.2.7) based on the data sheets in the tax_summary_a folder (relative abundance less than 2% was merged into “others”). The linear discriminant analysis effect size of samples (Guerrero-Preston et al., 2016) was based on different grouping conditions (Wilcoxon *P*-value < 0.05, logarithmic LDA (linear discriminant analysis) score > 2). PICRUSt (Tang et al., 2018) and FUNGuild (Fungi Functional Guild) (Schmidt et al., 2019) were used for the prediction of bacterial and fungal functions, respectively. Venn diagram analysis of operational taxonomic units (OTUs) (Ji et al., 2017) and canonical correlation analysis (Hu et al., 2017) were performed BASE and VEGAN packages in R (v.3.3.1), respectively.

The source values were defined according to (Xiong et al., 2021) with some modifications, where the transmission value is the number of OTUs shared by the target niche and the potential source niche/number of OTUs in the potential source niche; the known source value is the number of OTUs shared by the target niche and the potential source niche/number of OTUs in the target niche; and the unknown source value is the number of unique OTUs in the target niche/number of OTUs in the target niche.

## Results

### Microbiome assembly was more affected by compartment niches than developmental stages

**Figure 1:**
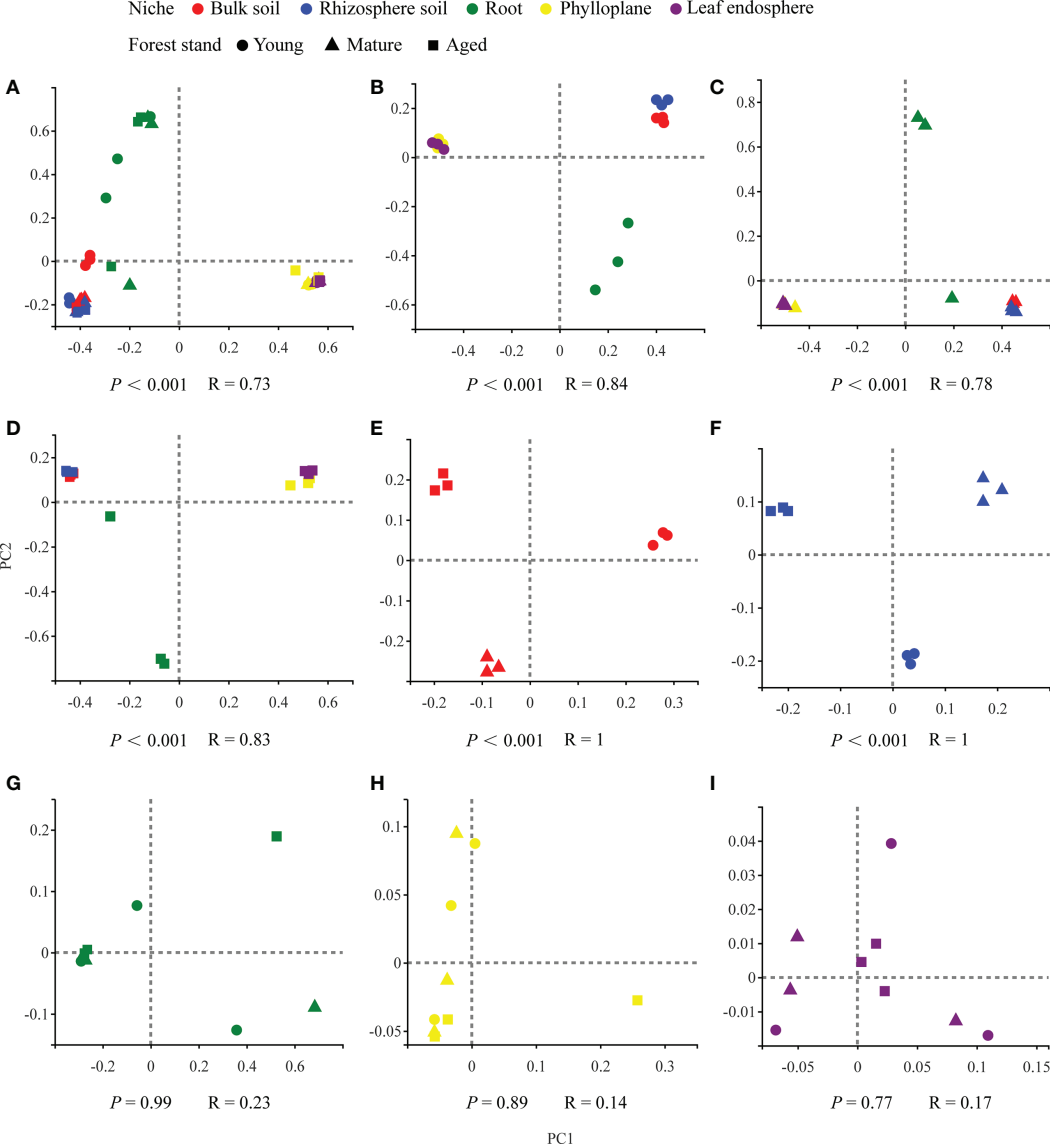
PCoA ordinations based on selected distance matrices of bacterial communities in each compartment niches from samples (*n* = 45). **(A)** all samples; **(B)** young forest stand; **(C)** mature forest stand; **(D)** aged forest stand; **(E)** bulk soil; **(F)** rhizosphere soil; **(G)** root; **(H)**phylloplane; **(I)** leaf endosphere.

**Figure 2:**
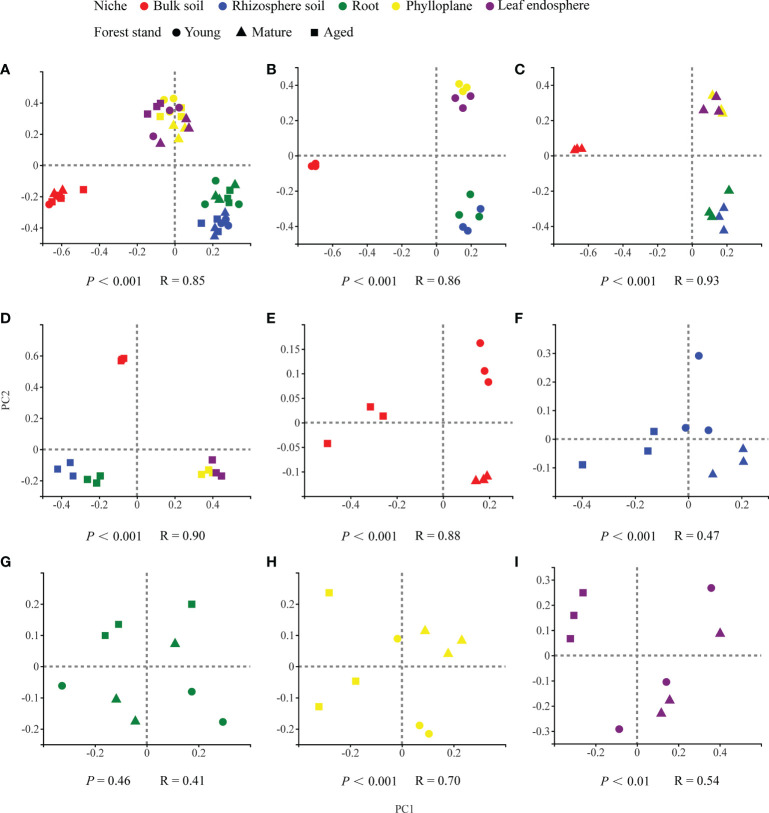
PCoA ordinations based on selected distance matrices of fungal communities in each compartment niches from samples (*n* = 45). **(A)** all samples; **(B)** young forest stand; **(C)** mature forest stand; **(D)** aged forest stand; **(E)** bulk soil; **(F)** rhizosphere soil; **(G)** root; **(H)** phylloplane; **(I)** leaf endosphere.

Our results based on the PCoA ordination of the complete dataset (*β*-diversity) showed that the microbiome assembly could be explained by different spatial ecological niches (**Figures 1B-D**; **Figures 2B-D**). Further studies on microbiomes in the same compartment niches at different developmental stages revealed that the bacterial and fungal microbiomes in soil were affected by the developmental stage (**Figures 1E, F**; **2E-F**); however, there were no significant differences in the bacterial community among the same plant compartment niches at different developmental stages (**Figures 1G-I**). Conversely, the fungal community in roots was not significantly affected by the developmental stage, whereas the effects of developmental stage were significant in the phyllosphere (**Figures 2G-I**). Moreover, as seen in **Figures 1A** and **2A**, there were clear and separate clustering among different compartment niches rather than developmental stages (bacteria: *P* < 0.001, R = 0.73; fungi: *P* < 0.001, R = 0.85). Differences in the microbial community were observed between the phyllosphere and rhizosphere. Thus, in the following analysis, we only focused on the effect of compartment niches on microbiomes.

### Microbial diversity and network complexity in compartment niches

**Figure 3:**
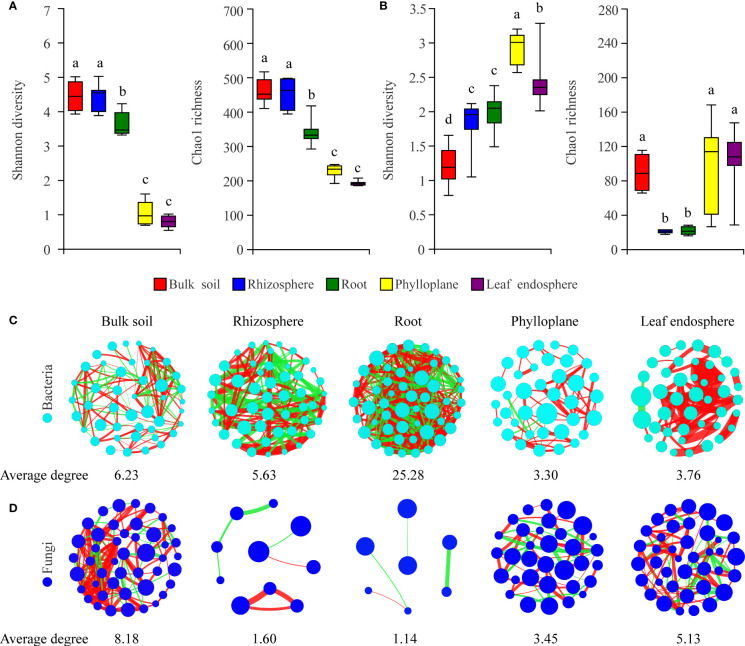
Compartment niches have a strong effect on microbial diversity and network complexity. **(A, C)** Bacterial diversity and co-occurrence networks along the soil–plant continuum based on all samples, respectively. **(B, D)** Fungal diversity and co-occurrence networks along the soil–plant continuum based on all samples, respectively. Different letters above the box plots indicate significant differences (*P* < 0.05). Average degree indicates the degree of network complexity among the microorganisms in the compartment niches. The size of nodes represents the species abundance. The color of the line indicates a positive (red) or negative (green) correlation. The thickness of the line indicates the magnitude of the correlation coefficient.

To characterize selection of the microbial community by *C*. *equisetifolia*, we assessed the *α*-diversity and co-occurrence patterns of the microbial communities along the soil–plant continuum. Our results showed that compartment niches had a strong effect on microbial diversity (Shannon diversity and Chao1 richness) and network complexity (with a higher average degree representing a greater network complexity) (**Figures 3A-D**). The bacterial Shannon diversity and Chao1 richness gradually decreased from the soils to roots to phyllosphere (**Figure 3A**); the highest network complexity was found in the roots (with an average degree of 25.28) and the lowest was found in the phylloplane (with an average degree of 3.30) (**Figure 3C**). However, the fungal Shannon diversity gradually increased from the soils to roots to phyllosphere, and the Chao1 richness decreased from the soils to roots but increased from the roots to phyllosphere (**Figure 3B**). The greatest network complexity was found in bulk soils (with an average degree of 8.18), whereas the lowest was found in the roots (with an average degree of 1.14) (**Figure 3D**).

### Assembly, keystone taxa, and biological functions of microbial communities in compartment niches

**Figure 4:**
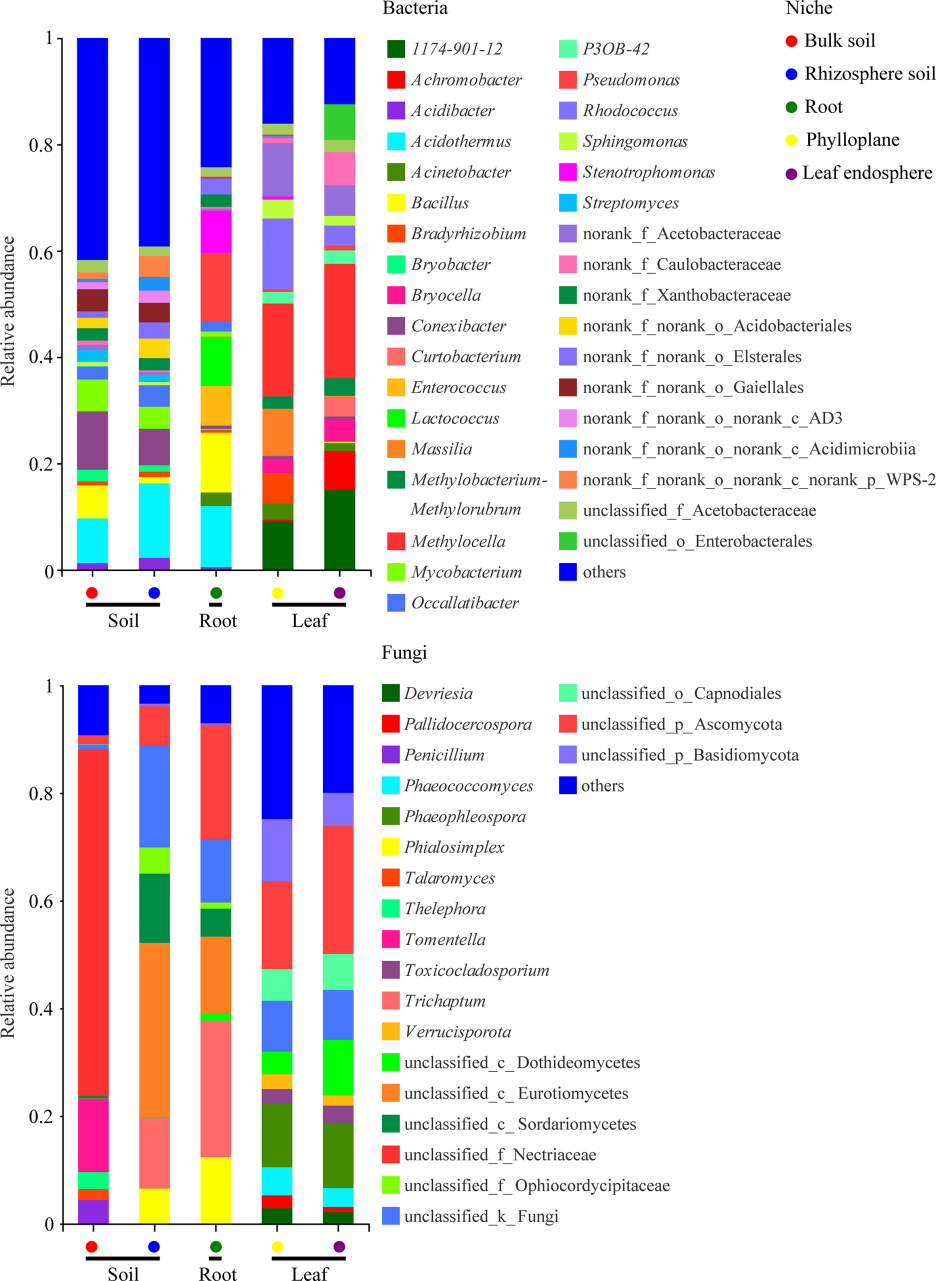
Relative abundances of the most prevalent bacterial (top) and fungal (bottom) genera in different compartment niches.

**Figure 5:**
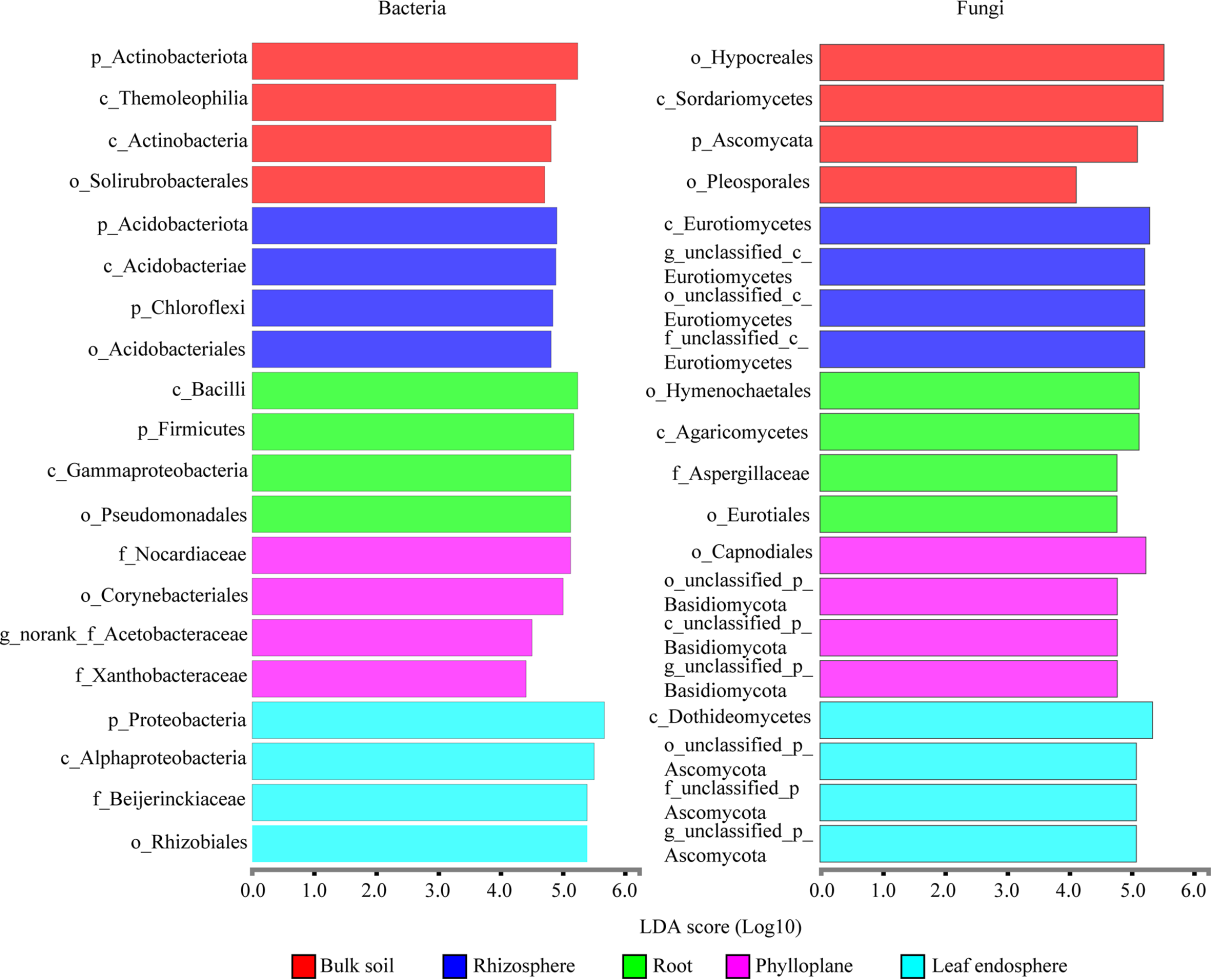
Linear discriminant analysis effect size identifying the keystone taxa associated with the different compartment niches (bacteria are on the left; fungi are on the right.). Only the top four most specific biomarker taxonomies are shown.

**Figure 6:**
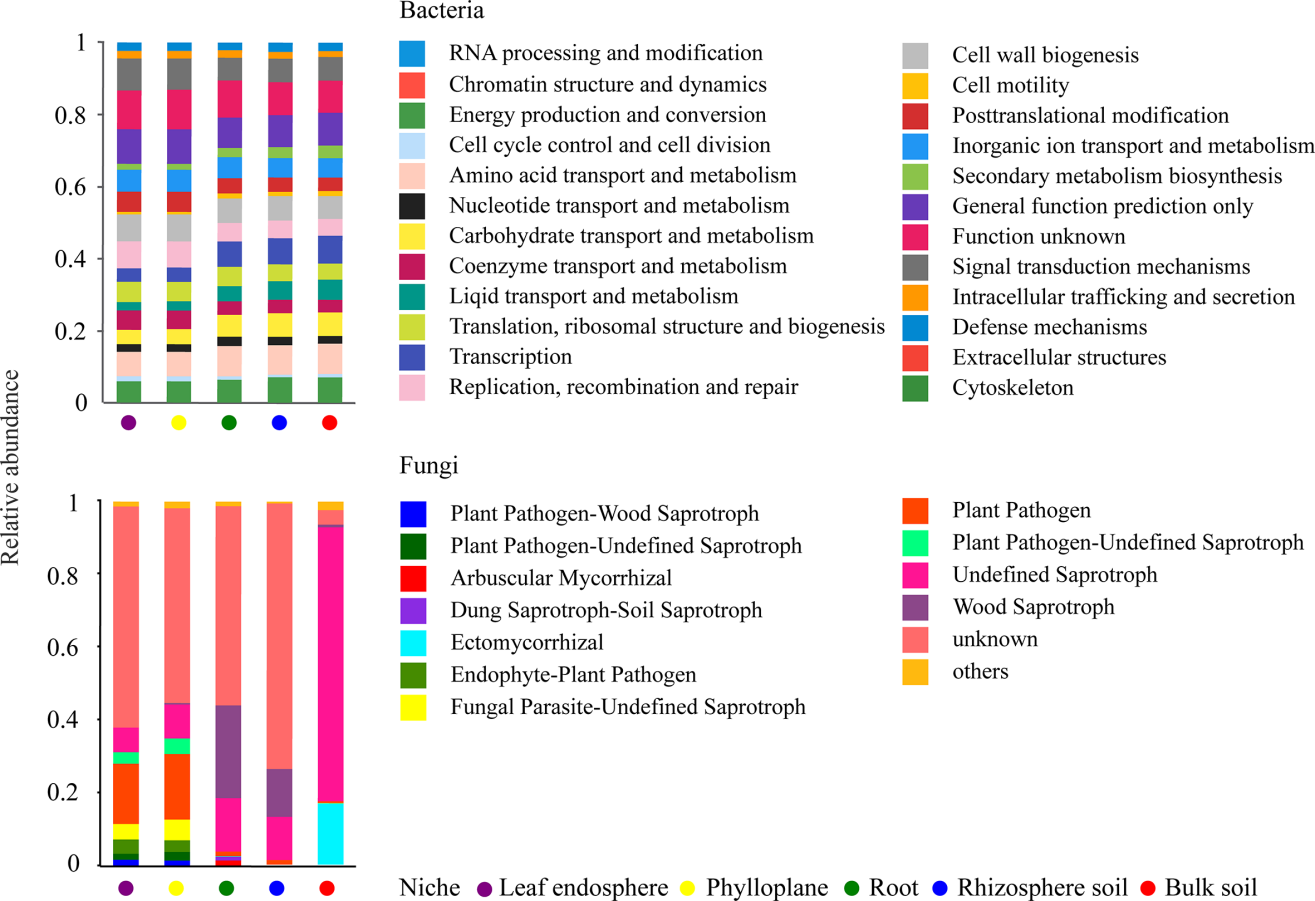
Potential functional groups of bacterial (top) and fungal (bottom) communities among different ecological niches.

A bar chart of the dominant bacterial communities in all samples (**Figure 4**) showed that bulk soil was dominated by *Conexibacter* (10.99%), rhizosphere soil by *Acidothermus* (14.04%), and the root endosphere by *Pseudomonas* (12.94%); *Methylocella* dominated both the phylloplane (17.46%) and leaf endosphere (21.49%). Regarding the fungal communities, bulk soil was dominated by unclassified_f_Nectriaceae (64.36%), rhizosphere soil by unclassified_c_Eurotiomycetes (32.38%), and the root endosphere by *Trichaptum* (25.35%); unclassified_p_Ascomycota dominated both the phylloplane (16.31%) and leaf endosphere (23.76%). The linear discriminant analysis effect size identified p_Actinobacteriota in bulk soils, p_Acidobacteriota in the rhizosphere, c_Bacilli in the roots, f_Nocardiaceae in the phylloplane, and p_Proteobacteria in the leaf endosphere as the most significant bacterial biomarker taxa, and o_Hypocreales in bulk soils, c_Eurotiomycetes in the rhizosphere, o_Hymenochaetales in the roots, o_Capnodiales in the phylloplane, and c_Dothideomycetes in the leaf endosphere as the most significant fungal keystone taxa (**Figure 5**). We identified 24 bacterial and 13 fungal functional groups (**Figure 6**). The functions of bacteria were mostly related to growth, metabolism, energy, and transport, whereas the functions of fungi mainly involved organic matter decomposition. The saprophytic and pathogenic microbes in the phyllosphere exhibited substantial abundance and diversity, with the saprophytes being most abundant in the soil.

### Sources of microorganisms in different ecological niches

**Figure 7:**
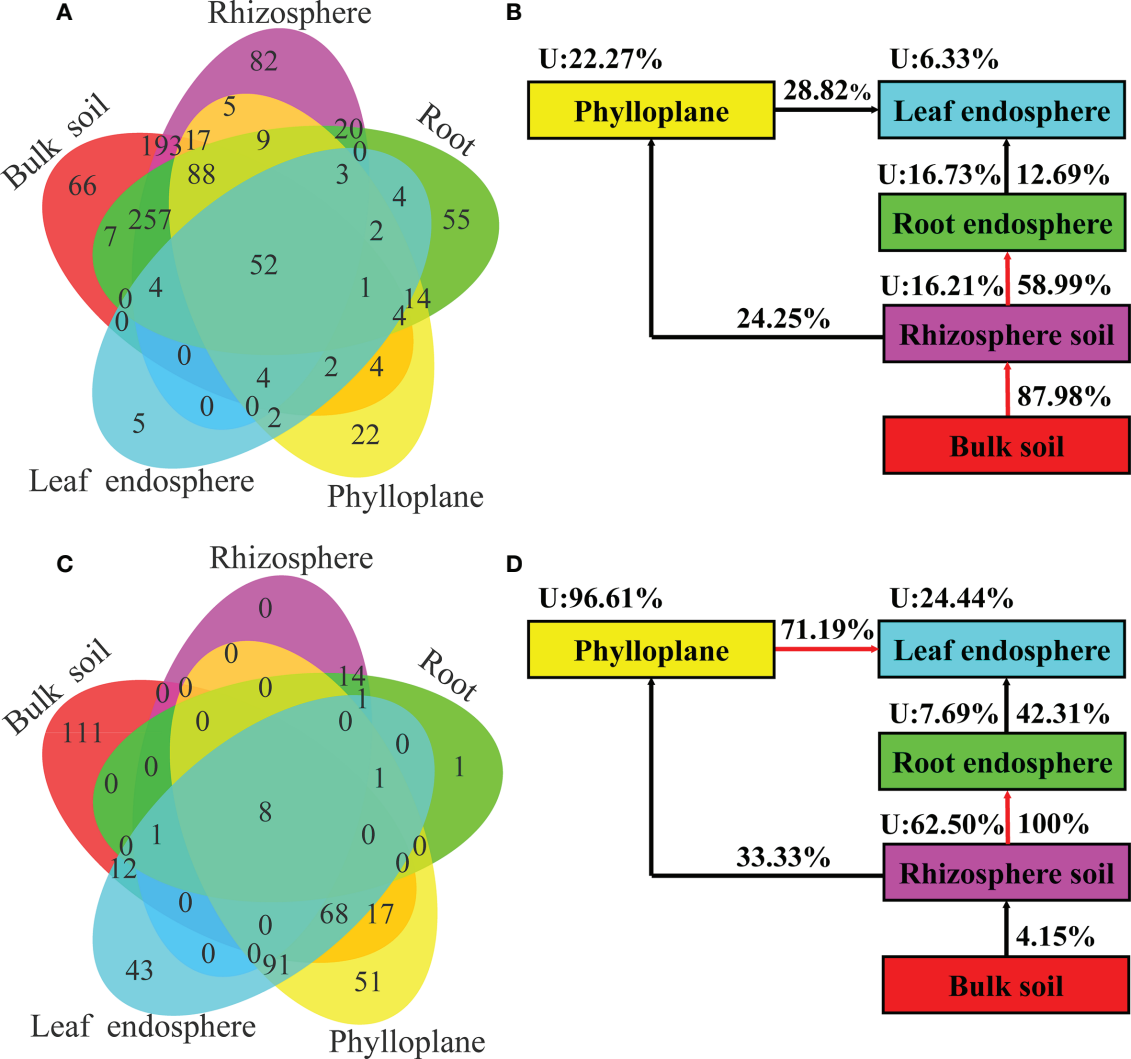
**(A, C)** Venn diagram of bacterial (top) and fungal (bottom) microbiomes showing the potential sources based on all samples. **(B, D)** Simplified version of **(A, C)**, respectively. U represents the unknown source. The red arrow represents a spread value greater than 50%.

A Venn diagram based on bacterial OTUs at the genus level was constructed to identify the potential sources of obverted microbial communities in each compartment niche. It revealed that the bacterial communities in *C*. *equisetifolia* mainly originated from bulk soil and were sequentially filtered by the plant niches (**Figures 7A, B**). However, the fungal communities in *C*. *equisetifolia* were mainly derived from rhizosphere soil and air (**Figures 7C, D**). Most taxa in the nearby microbial sources were selected by the root endosphere and leaf endosphere, with an unknown source value < 25%. The unknown source value of the fungal community in the phylloplane was higher (96.61%) than that of the bacterial community in the phylloplane.

### Effects of environmental factors on microorganisms of different ecological niches

**Figure 8:**
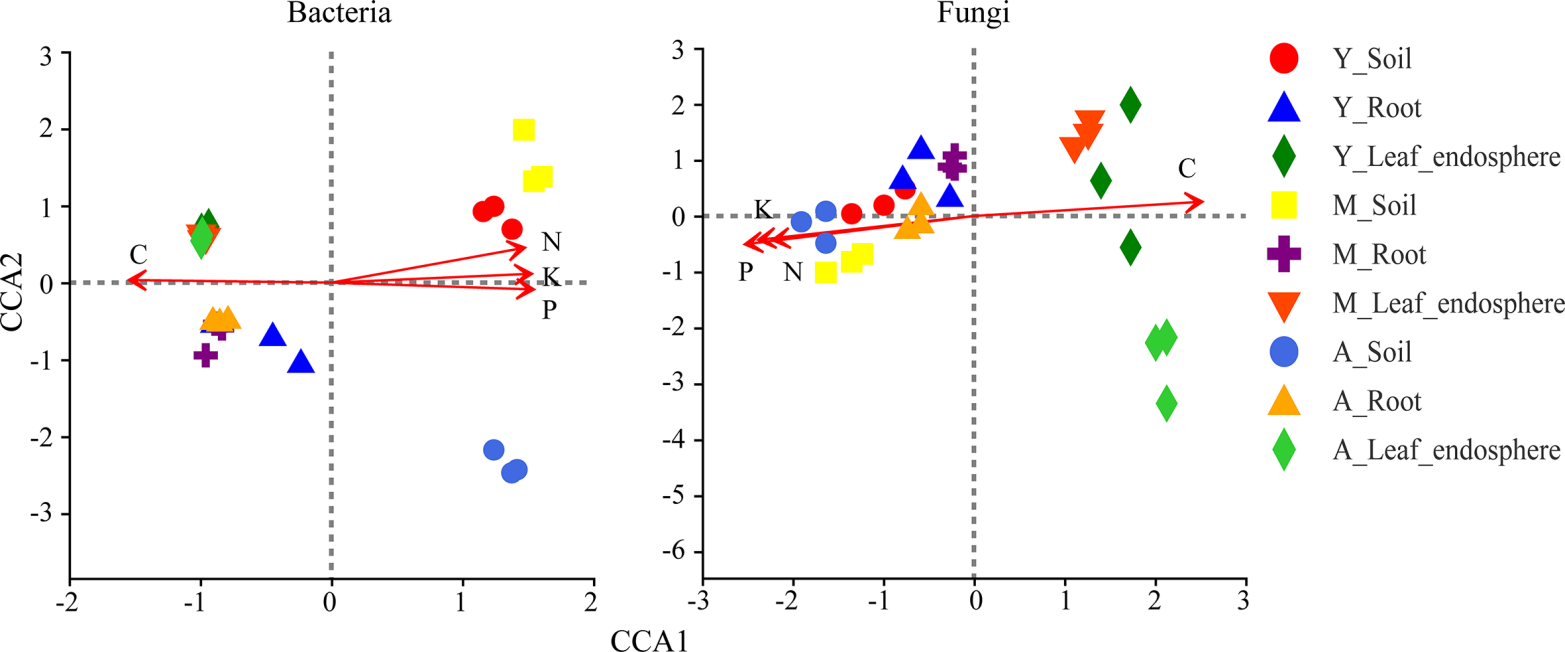
Canonical correlation analysis of bacteria (left) and fungi (right) genera in different niches in the genus stand. Red arrow represents environmental factors, and the length of the arrow represents the degree of impact of environmental factors on species data. Angle between the arrows of environmental factors represents positive and negative correlations (acute angle: positive correlation; obtuse angle: negative correlation; right angle: no correlation). Sample points were projected onto the arrows of quantitative environmental factors, and the distance between the projection points and the origin represents the relative influence of environmental factors on the distribution of sample communities. C, carbon; N, nitrogen; P, phosphorus; K, kalium.

As seen in **Table 1**, the organic carbon content was higher in the leaf and root than the soil, whereas the nitrogen, phosphorus, and potassium contents were greatest in the soil. **Figure 8** shows that organic carbon was negatively correlated with total nitrogen, total phosphorus, and total potassium. Leaf microorganisms were positively correlated with organic carbon, and root and soil microorganisms were positively correlated with total nitrogen, total phosphorus, and total potassium. The relationship between microorganisms and these chemicals depended mainly on the compartment niche rather than the developmental stage.

**Table 1:** Relative contents of total carbon, nitrogen, phosphorus, and potassium in different ecological niches.

	C	N	P	K
Niche	Relative content (g/kg)	Relative content (g/kg)	Relative content (g/kg)	Relative content (g/kg)
Y Leaf endosphere	162.88 ± 6.11 bc	24.73 ± 1.75 c	0.89 ± 0.06 d	3.84 ± 0.01 d
M Leaf endosphere	159.58 ± 2.97 c	25.06 ± 0.70 c	1.50 ± 0.04 d	12.60 ± 0.64 d
A Leaf endosphere	175.63 ± 1.36 a	17.91 ± 2.94 c	1.16 ± 0.13 d	5.47 ± 0.95 d
Y Root	167.84 ± 1.03 abc	8.84 ± 0.47 c	0.64 ± 0.02 d	3.12 ± 0.26 d
M Root	162.57 ± 3.84 bc	7.01 ± 0.57 c	0.40 ± 0.05 d	1.63 ± 0.20 d
A Root	171.04 ± 2.08 ab	6.96 ± 0.23 c	0.35 ± 0.10 d	0.96 ± 0.11 d
Y Soil	3.73 ± 0.06 d	435.65 ± 6.79 a	498.63 ± 3.06 c	168.10 ± 8.80 c
M Soil	8.19 ± 0.19 d	418.32 ± 18.16 a	552.76 ± 4.07 b	156.50 ± 4.61 b
A Soil	4.25 ± 0.27 d	239.91 ± 13.86 b	573.10 ± 0.27 a	140.63 ± 3.40 a

The value is the average of each group, and the unit is grams per kilogram. Y, young; M, mature; A, aged.

Different letters indicate significant differences (P < 0.05).

## Discussion

While the diversity of plant-associated microbiomes is increasingly recognized, we still need to further clarify the relationship between microbiomes and their host plant. In this study, we investigated the microbial communities along the soil–plant continuum of *C*. *equisetifolia* at different forest ages. We found that the structure and diversity of microbial communities were shaped by compartment niches. Our results further showed that bacterial diversity and richness decreased from the soils to roots to leaves, with the highest network complexity found in the roots and the lowest in the phylloplane. However, fungal diversity gradually increased from the soils to roots to phyllosphere, whereas richness decreased from the soils to roots but increased from the roots to phyllosphere; the greatest network complexity was found in bulk soils and the lowest was found in the roots. These findings provide comprehensive and empirical evidence for theories regarding non-crop host selection and niche occupation involved in *C*. *equisetifolia* microbiome assembly. In addition, we identified the dominant taxa, ecological functions, and the potential sources as well as the effects of environmental factors on non-crop microbiomes. Our results provide key information for non-crop microbiome manipulation.

### Assembly and diversity of microbial communities in compartment niches

In our study, we found clear and separate clustering among different compartment niches rather than among developmental stages (**Figures 1A**, **2A**). Thus, we inferred that the *C*. *equisetifolia* microbiome assemblage was predominantly shaped by the compartment niches, as suggested by previous studies (Beckers et al., 2017; Cregger et al., 2018). We also found that the bacterial Shannon diversity decreased from the soil to roots and then to the leaves, whereas the fungal diversity showed the opposite trend (**Figures 3A, B**), which might be attributed to the external environment, host plant, and microbial properties (Edwards et al., 2015; Hamonts et al., 2018). These results suggest significant differences in the assembly of bacteria and fungi across compartment niches.

We further found that the lowest Chao1 richness, Shannon diversity, and network complexity of bacteria occurred in the phyllosphere (**Figures 3A, C**). A leaf environment with low bacterial diversity is considered unfavorable for bacterial colonization because the leaf surface is exposed to rapidly changing temperature and humidity, as well as the alternating presence and absence of rainwater, which can reduce the bacterial diversity (Guttman et al., 2014). Moreover, leaves provide limited nutrients for bacteria, and you could find less competition. As a result, most bacteria that migrate to leaves may find themselves in a nutrient-poor environment that limits their growth and metabolism (Kembel et al., 2014; Hacquard et al., 2015). Interestingly, we found that leaves harbored a diverse range of fungal taxa (**Figure 3B**), probably because fungi are typically more tolerant to drought and can proliferate in harsh environments, enabling better survival of the fungal community in above-ground plant tissues (Whipps et al., 2008; Rodriguez et al., 2009). Hainan Province has a large diurnal temperature range and distinct seasons with abundant and deficient rainfall, with drought typically occurring in the season with low rainfall (Cai et al., 2010). Moreover, *C*. *equisetifolia* bears coriaceous scale leaves, and photosynthesis mainly occurs in needle-like branchlets, which leads to low water retention. These factors may contribute to the harsh microenvironment of *C*. *equisetifolia* leaves.

We also found that bacterial diversity in the rhizosphere soil was consistently higher than that in the root endosphere (**Figure 3A**). This is not surprising because the root exudates produced by *C*. *equisetifolia* in the rhizosphere (e.g., 2,4-di-tert-butylphenol, methyl stearate, and arginine) enhance the bacterial chemoattraction and colonization of the rhizosphere soil and rhizoplane. These factors lead to the formation of a unique, highly abundant, and diverse microbial community in the rhizosphere (Lin, unpublished data). After colonization at the rhizoplane, soil bacteria compatible with the plant lifestyle will reach the xylem vessels by active or passive transport through the endodermis and pericycle. The rhizoplane acts as a selective barrier responsible for a considerable loss of microbial diversity (Hardoim et al., 2008). *Via* a systematic evaluation of the relative contribution of various niches to the microbial community, our work enhances the understanding of plant microbiome assembly.

### Dominant taxa and biological functions of microbial communities in compartment niches

Dominant taxa can be key microbes with important ecological implications in microbiome assembly and ecosystem functioning (Banerjee et al., 2018; Delgado-Baquerizo et al., 2018). Our results indicated that the dominant taxa in the root endosphere were *Pseudomonas* (Gammaproteobacteria) and *Bacillus* (Bacilli) (**Figure 4**). Members of Gammaproteobacteria can colonize the rhizosphere and a wide range of niches, thereby playing a key role in regulating host fitness, pathogen inhibition, and plant tolerance (Mendes et al., 2011; Álvarez-Pérez et al., 2017). We further found that *Pseudomonas* was more abundant in the roots than in the soils (**Figure 4**) because, although found in the rhizosphere where it greatly promotes plant growth, *Pseudomonas* has poor environmental adaptability and low competitiveness (Berg, 2009). Therefore, we speculated that *Pseudomonas* could be more adapted to the root endosphere than to the soil, which explains their dominance in this environment. *Pseudomonas* is also growth-promoting bacteria capable of nitrogen-fixing and phosphorus dissolution (Berg, 2009). Bacilli are frequently reported as antagonistic bacteria against soil-borne diseases. Moreover, members of *Bacillus* are repeatedly shown to be growth-promotors living in the rhizosphere and capable of phosphorus solubilization and nitrogen-fixing (Gong et al., 2014). Our results indicated that the nitrogen content was higher than the potassium content in the roots (**Table 1**), which might be related to nitrogen-fixing by *Pseudomonas* and *Bacillus*; however, the fact that the phosphorus content was relatively lower requires further study.

Our results also revealed that Dothideomycetes were present in the phyllosphere (**Figure 4**), which agrees with the findings of (Adams et al., 2013) and suggests that Dothideomycetes may be airborne. In addition, many members of Dothideomycetes are saprophytic fungi associated with litter decomposition and nutrient cycling (Adams et al., 2013; Hyde et al., 2013). In the present study, most fungi were saprophytes enriched in the leaves (**Figure 6**), suggesting that the occupation of aged host plants by saprophytic fungi resulted from reduced immunity and the increasingly important ecological functions of saprophytic fungi as decomposers. As a result, we speculated that the adaptability of *C*. *equisetifolia* was improved by the growth and proliferation of probiotic microorganisms with various functions and living in different niches, which also supported the low soil fertility tolerance of this plant.

Together, these results suggest that plants can recruit microbial taxa with specific functions and adaptability in various compartment niches (Foster et al., 2017; Cordovez et al., 2019). Identification of these dominant taxa provides essential information for the development of strategies to manipulate the microbial community in *C*. *equisetifolia*.

### Sources of microbial communities in different ecological niches

Identifying the potential sources and enrichment processes of microbial communities in *C*. *equisetifolia* is essential for understanding the interactions among plants, soil, and microorganisms. Although previous studies have reported that the above-ground and below-ground compartments of plants share a large proportion of microbial taxa (Bai et al., 2015), little is known about enrichment of the microbiome in *C*. *equisetifolia*. Our results showed that the bacterial community in rhizosphere soil was mainly derived from bulk soil (unknown source values < 17%) and was sequentially filtered by the plant niches (**Figures 7A, B**). This was expected because the rhizoplane acts as a selective barrier. Meanwhile, limited bacterial species can colonize the root endosphere, such as those that express chemotaxis-related genes, present the formation of flagella and pellets, produce cell wall-degrading enzymes, and have complex interactions with the host plant immune system. Thus, the diversity of endophytic bacteria was lower than that of soil bacteria (Hardoim et al., 2008; Bulgarelli et al., 2012).

Moreover, although a small proportion of fungal OTUs were shared between the phylloplane and rhizosphere soil, more than 96% of fungi were of unknown sources (**Figures7C, D**). Therefore, we inferred that phylloplane fungi were mainly derived from the surrounding environment, suggesting air, dust, and rainwater as the primary sources of the phylloplane fungal community. We also found that leaf endosphere fungi originated from the phylloplane and soil (**Figures  7C, D**), and the roots may serve as an important transition boundary (Hacquard et al., 2015; Vandenkoornhuyse et al., 2015), allowing rhizosphere microbes to enter plant tissues and migrate to the above-ground plant compartments. In the phyllosphere, the unknown source values of bacteria were lower than those of fungi (**Figure 7**), indicating that a greater proportion of bacterial communities in the above-ground plant tissues was derived from rhizosphere soil. In addition, the unknown source values of leaf endophytes were lower than those of leaf epiphytes (**Figure 7**), further highlighting the selection of endophytes by hosts. These findings have identified potential sources and driving forces of microbial communities in the phyllosphere. Furthermore, they further confirm the phylloplane and rhizoplane as important interfaces among hosts, microorganisms, and the environment (Lindow and Brandl, 2003; Vorholt, 2012; Vacher et al., 2016; Remus-Emsermann and Schlechter, 2018).

In summary, our research showed that plants can recruit microbial taxa with specific function and niche adaptability, which provides a theoretical basis for the analysis of plant niches and transmission routes of microorganisms. However, the molecular mechanisms by which hosts regulate plant–microbiome interactions and microbial community dispersal are not fully understood; thus, further research is required.

## Conclusions

In this study, we provide comprehensive and empirical evidence for the relative contribution of compartment niches to microbiome assembly in *C*. *equisetifolia*. Our results suggest that microbiome assemblages along the soil–plant continuum were primarily shaped by the compartment niches rather than the developmental stage. Moreover, bacterial diversity and richness decreased from the soils to roots to leaves, with the highest network complexity found in the roots and the lowest in the phylloplane. However, fungal diversity gradually increased from the soils to roots to phyllosphere, whereas richness decreased from the soils to roots and increased from the roots to phyllosphere; the greatest network complexity was found in bulk soils, and the lowest was in the roots. Furthermore, different biomarker taxa were present in different ecological niches, and significant differences in ecological function were found between bacterial and fungal communities, with bacteria playing an important role in maintaining plant growth and providing nutrients, whereas fungi played a dominant role in the decomposition of organic matter. In addition, the bacterial community of *C*. *equisetifolia* was mainly derived from bulk soil, whereas the fungal community primarily originated from the rhizosphere soil and air, with leaf microorganisms positively correlated with organic carbon, and root and soil microorganisms positively correlated with total nitrogen, total phosphorus, and total potassium. These results suggest strong selective and regulatory effects of plant hosts on the composition and potential function of plant microbial communities. The results of this study have implications for future non-crop management by providing baseline data to inform translational research into harnessing the plant microbiome.

## Data availability statement

The original contributions presented in the study are included in the article/supplementary material. Further inquiries can be directed to the corresponding author.

## Author contributions

LL conceived the idea and designed the experiment; QL performed the experiment, analyzed the data, and wrote the paper; YW, ML, and ZX performed the experiment. All authors contributed to the article and approved the submitted version.

## Funding

This work was supported by the innovation platform for academicians of Hainan Province (YSPTZX202129) and the Youth Science and Technology Talents Innovation Program of Hainan Association for Science and Technology (QCXM201905).

## Acknowledgments

We thank Prof. Rodolfo Dirzo from the Department of Biology Stanford University for their kind advice on data processing and manuscript writing. We thank Rui Huang for the field trips.

## Conflict of interest

The authors declare that the research was conducted in the absence of any commercial or financial relationships that could be construed as a potential conflict of interest.

## Publisher’s note

All claims expressed in this article are solely those of the authors and do not necessarily represent those of their affiliated organizations, or those of the publisher, the editors and the reviewers. Any product that may be evaluated in this article, or claim that may be made by its manufacturer, is not guaranteed or endorsed by the publisher.

## References

[B1] Abdelfattah A. Wisniewski M. Schena L. Tack A. J. M. (2021). Experimental evidence of microbial inheritance in plants and transmission routes from seed to phyllosphere and root. Environ. Microbiol. 23, 2199–2214. doi: 10.1111/1462-2920.15392 33427409

[B2] Adams R. I. Miletto M. Taylor J. W. Bruns T. D. (2013). Dispersal in microbes: Fungi in indoor air are dominated by outdoor air and show dispersal limitation at short distances. Isme J. 7, 1262–1273. doi: 10.1038/ismej.2013.28 23426013 PMC3695294

[B3] Álvarez-Pérez J. M. González-García S. Cobos R. Olego M. Ibañez A. Díez-Galán A.. (2017). Use of endophytic and rhizosphere actinobacteria from grapevine plants to reduce nursery fungal graft infections that lead to young grapevine decline. Appl. Environ. Microbiol. 83, e01564–e01517. doi: 10.1128/aem.01564-17 28986378 PMC5717199

[B4] Bai Y. Müller D. B. Srinivas G. Garrido-Oter R. Potthoff E. Rott M.. (2015). Functional overlap of the *arabidopsis* leaf and root microbiota. Nature 528, 364–369. doi: 10.1038/nature16192 26633631

[B5] Banerjee S. Schlaeppi K. van der Heijden M. G. A. (2018). Keystone taxa as drivers of microbiome structure and functioning. Nat. Rev. Microbiol. 16, 567–576. doi: 10.1038/s41579-018-0024-1 29789680

[B6] Beckers B. Op De Beeck M. Weyens N. Boerjan W. Vangronsveld J. (2017). Structural variability and niche differentiation in the rhizosphere and endosphere bacterial microbiome of field-grown poplar trees. Microbiome 5, 25. doi: 10.1186/s40168-017-0241-2 28231859 PMC5324219

[B7] Berg G. (2009). Plant-microbe interactions promoting plant growth and health: Perspectives for controlled use of microorganisms in agriculture. Appl. Microbiol. Biotechnol. 84, 11–18. doi: 10.1007/s00253-009-2092-7 19568745

[B8] Bodenhausen N. Horton M. W. Bergelson J. (2013). Bacterial communities associated with the leaves and the roots of *arabidopsis thaliana* . PloS One 8, e56329. doi: 10.1371/journal.pone.0056329 23457551 PMC3574144

[B9] Bulgarelli D. Rott M. Schlaeppi K. Ver Loren van Themaat E. Ahmadinejad N. Assenza F.. (2012). Revealing structure and assembly cues for *arabidopsis* root-inhabiting bacterial microbiota. Nature 488, 91–95. doi: 10.1038/nature11336 22859207

[B10] Bulgarelli D. Schlaeppi K. Spaepen S. Ver Loren van Themaat E. Schulze-Lefert P. (2013). Structure and functions of the bacterial microbiota of plants. Annu. Rev. Plant Biol. 64, 807–838. doi: 10.1146/annurev-arplant-050312-120106 23373698

[B11] Cai D. Liu S. Tian G. Xu X. Cui D. Zhang J. (2010). Analysis of tourism climate resources in hainan island. Modern Agric. Technol., 18–20+24. Available at: https://kns.cnki.net/kcms/detail/detail.aspx?FileName=ANHE201016006&DbName=CJFQ2010

[B12] Calderón K. Spor A. Breuil M. C. Bru D. Bizouard F. Violle C.. (2017). Effectiveness of ecological rescue for altered soil microbial communities and functions. Isme J. 11, 272–283. doi: 10.1038/ismej.2016.86 27341455 PMC5315478

[B13] Caporaso J. G. Kuczynski J. Stombaugh J. Bittinger K. Bushman F. D. Costello E. K.. (2010). Qiime allows analysis of high-throughput community sequencing data. Nat. Methods 7, 335–336. doi: 10.1038/nmeth.f.303 20383131 PMC3156573

[B14] Chen S. Zhou Y. Chen Y. Gu J. (2018). Fastp: An ultra-fast all-in-one fastq preprocessor. Bioinformatics 34, i884–i890. doi: 10.1093/bioinformatics/bty560 30423086 PMC6129281

[B15] Compant S. Cambon M. C. Vacher C. Mitter B. Samad A. Sessitsch A. (2021). The plant endosphere world - bacterial life within plants. Environ. Microbiol. 23, 1812–1829. doi: 10.1111/1462-2920.15240 32955144

[B16] Cordovez V. Dini-Andreote F. Carrión V. J. Raaijmakers J. M. (2019). Ecology and evolution of plant microbiomes. Annu. Rev. Microbiol. 73, 69–88. doi: 10.1146/annurev-micro-090817-062524 31091418

[B17] Cregger M. A. Veach A. M. Yang Z. K. Crouch M. J. Vilgalys R. Tuskan G. A.. (2018). The *populus* holobiont: Dissecting the effects of plant niches and genotype on the microbiome. Microbiome 6, 31. doi: 10.1186/s40168-018-0413-8 29433554 PMC5810025

[B18] Delgado-Baquerizo M. Oliverio A. M. Brewer T. E. Benavent-González A. Eldridge D. J. Bardgett R. D.. (2018). A global atlas of the dominant bacteria found in soil. Science 359, 320–325. doi: 10.1126/science.aap9516 29348236

[B19] Edgar R. C. (2013). Uparse: Highly accurate otu sequences from microbial amplicon reads. Nat. Methods 10, 996–998. doi: 10.1038/nmeth.2604 23955772

[B20] Edwards J. Johnson C. Santos-Medellín C. Lurie E. Podishetty N. K. Bhatnagar S.. (2015). Structure, variation, and assembly of the root-associated microbiomes of rice. Proc. Natl. Acad. Sci. U.S.A. 112, E911–E920. doi: 10.1073/pnas.1414592112 25605935 PMC4345613

[B21] Foster K. R. Schluter J. Coyte K. Z. Rakoff-Nahoum S. (2017). The evolution of the host microbiome as an ecosystem on a leash. Nature 548, 43–51. doi: 10.1038/nature23292 28770836 PMC5749636

[B22] Gong R. Wang X. Zhao J. Zhang S. Wang R. Meng J. (2014). Isolation and identification of phosphorus - solubilizing bacteria from rhizosphere of desert steppe plants and comparison of their phosphorus-solubilizing abilities. Inner Mongolia Agric. Sci. Technol. 26, 6–9. doi: 10.3969/j.issn.1007-0907.2014.04.003

[B23] Guerrero-Preston R. Godoy-Vitorino F. Jedlicka A. Rodríguez-Hilario A. González H. Bondy J.. (2016). 16s rrna amplicon sequencing identifies microbiota associated with oral cancer, human papilloma virus infection and surgical treatment. Oncotarget 7, 51320–51334. doi: 10.18632/oncotarget.9710 27259999 PMC5239478

[B24] Guttman D. S. McHardy A. C. Schulze-Lefert P. (2014). Microbial genome-enabled insights into plant-microorganism interactions. Nat. Rev. Genet. 15, 797–813. doi: 10.1038/nrg3748 25266034

[B25] Hacquard S. Garrido-Oter R. González A. Spaepen S. Ackermann G. Lebeis S.. (2015). Microbiota and host nutrition across plant and animal kingdoms. Cell Host Microbe 17, 603–616. doi: 10.1016/j.chom.2015.04.009 25974302

[B26] Hamonts K. Trivedi P. Garg A. Janitz C. Grinyer J. Holford P.. (2018). Field study reveals core plant microbiota and relative importance of their drivers. Environ. Microbiol. 20, 124–140. doi: 10.1111/1462-2920.14031 29266641

[B27] Hardoim P. R. van Overbeek L. S. Elsas J. D. (2008). Properties of bacterial endophytes and their proposed role in plant growth. Trends Microbiol. 16, 463–471. doi: 10.1016/j.tim.2008.07.008 18789693

[B28] Huang R. (2019). Root endophytic bacteria diversity and allelopathic potential of their metabolites in casuarina equisetifolia at different ages (Hainan Normal University). Available at: https://kns.cnki.net/KCMS/detail/detail.aspx?dbname=CMFD202001&filename=1019220682.nh

[B29] Hu H. Chen X. Hou F. Wu Y. Cheng Y. (2017). Bacterial and fungal community structures in loess plateau grasslands with different grazing intensities. Front. Microbiol. 8, 606. doi: 10.3389/fmicb.2017.00606 28439265 PMC5383705

[B30] Hyde K. Jones E. Liu J. Ariyawansa H. Boehm E. Boonmee S. (2013). Families of dothideomycetes. Fungal Diversity 63, 1–313. doi: 10.1007/s13225-013-0263-4

[B31] Ji P. Rhoads W. J. Edwards M. A. Pruden A. (2017). Impact of water heater temperature setting and water use frequency on the building plumbing microbiome. Isme J. 11, 1318–1330. doi: 10.1038/ismej.2017.14 28282040 PMC5437349

[B32] Kembel S. W. O'Connor T. K. Arnold H. K. Hubbell S. P. Wright S. J. Green J. L. (2014). Relationships between phyllosphere bacterial communities and plant functional traits in a neotropical forest. Proc. Natl. Acad. Sci. U.S.A. 111, 13715–13720. doi: 10.1073/pnas.1216057111 25225376 PMC4183302

[B33] Li Q. Mo Q. Wang F. Li Y. Xu X. Zou B.. (2015). Nutrient utilization characteristics of *casuarina equisetifolia* plantations of different ages in tropical coastal south china. Chin. J. Appl. Environ. Biol. 21, 139–146. doi: 10.3724/SP.J.1145.2014.07001

[B34] Lindow S. E. Brandl M. T. (2003). Microbiology of the phyllosphere. Appl. Environ. Microbiol. 69, 1875–1883. doi: 10.1128/AEM.69.4.1875-1883.2003 12676659 PMC154815

[B35] Lin W. X. Ye G. F. Tan F. L. Nie S. XU J. S. (2008). Fractal feature of soil structure and reflection on soil properties in different regeneration patterns of *casuarina equisetifolia* forest in coastal sandy soil. Chin. J. Eco-Agric. 16, 1352–1357. doi: 10.3724/SP.J.1011.2008.01352

[B36] Liu H. Li K. Hong T. Fan H. Lin Y. Li J.. (2020). Root morphological characteristics of wild seedlings under *casuarina equisetifolia* forest in changle coastal fuzhou. J. Trop. Crops 41, 2555–2561. doi: 10.3969/j.issn.1000-2561.2020.12.026

[B37] Magoč T. Salzberg S. L. (2011). Flash: Fast length adjustment of short reads to improve genome assemblies. Bioinformatics 27, 2957–2963. doi: 10.1093/bioinformatics/btr507 21903629 PMC3198573

[B38] Mendes R. Kruijt M. de Bruijn I. Dekkers E. van der Voort M. Schneider J. H.. (2011). Deciphering the rhizosphere microbiome for disease-suppressive bacteria. Science 332, 1097–1100. doi: 10.1126/science.1203980 21551032

[B39] Müller D. B. Vogel C. Bai Y. Vorholt J. A. (2016). The plant microbiota: Systems-level insights and perspectives. Annu. Rev. Genet. 50, 211–234. doi: 10.1146/annurev-genet-120215-034952 27648643

[B40] Ramayo-Caldas Y. Mach N. Lepage P. Levenez F. Denis C. Lemonnier G.. (2016). Phylogenetic network analysis applied to pig gut microbiota identifies an ecosystem structure linked with growth traits. Isme J. 10, 2973–2977. doi: 10.1038/ismej.2016.77 27177190 PMC5148198

[B41] Remus-Emsermann M. N. P. Schlechter R. O. (2018). Phyllosphere microbiology: At the interface between microbial individuals and the plant host. New Phytol. 218, 1327–1333. doi: 10.1111/nph.15054 29504646

[B42] Ren F. Dong W. Yan D. H. (2019). Organs, cultivars, soil, and fruit properties affect structure of endophytic mycobiota of pinggu peach trees. Microorganisms 7, 322. doi: 10.3390/microorganisms7090322 PMC6780621 31492017

[B43] Rodriguez R. J. White J. F. Jr. Arnold A. E. Redman R. S. (2009). Fungal endophytes: Diversity and functional roles. New Phytol. 182, 314–330. doi: 10.1111/j.1469-8137.2009.02773.x 19236579

[B44] Rogers M. B. Firek B. Shi M. Yeh A. Brower-Sinning R. Aveson V.. (2016). Disruption of the microbiota across multiple body sites in critically ill children. Microbiome 4, 66. doi: 10.1186/s40168-016-0211-0 28034303 PMC5200963

[B45] Schmidt R. Mitchell J. Scow K. (2019). Cover cropping and no-till increase diversity and symbiotroph:Saprotroph ratios of soil fungal communities. Soil Biol. Biochem. 129, 99–109. doi: 10.1016/j.soilbio.2018.11.010

[B46] Tang R. Wei Y. Li Y. Chen W. Chen H. Wang Q.. (2018). Gut microbial profile is altered in primary biliary cholangitis and partially restored after udca therapy. Gut 67, 534–541. doi: 10.1136/gutjnl-2016-313332 28213609

[B47] Vacher C. Hampe A. Porté A. Sauer U. Compant S. Morris C. (2016). The phyllosphere: Microbial jungle at the plant–climate interface. Annu. Rev. Ecol. Evol. S. 47, 1–24. doi: 10.1146/annurev-ecolsys-121415-032238

[B48] Vandenkoornhuyse P. Quaiser A. Duhamel M. Le Van A. Dufresne A. (2015). The importance of the microbiome of the plant holobiont. New Phytol. 206, 1196–1206. doi: 10.1111/nph.13312 25655016

[B49] Vorholt J. A. (2012). Microbial life in the phyllosphere. Nat. Rev. Microbiol. 10, 828–840. doi: 10.1038/nrmicro2910 23154261

[B50] Whipps J. M. Hand P. Pink D. Bending G. D. (2008). Phyllosphere microbiology with special reference to diversity and plant genotype. J. Appl. Microbiol. 105, 1744–1755. doi: 10.1111/j.1365-2672.2008.03906.x 19120625

[B51] Xiong C. Zhu Y. G. Wang J. T. Singh B. Han L. L. Shen J. P.. (2021). Host selection shapes crop microbiome assembly and network complexity. New Phytol. 229, 1091–1104. doi: 10.1111/nph.16890 32852792

[B52] Zhang J. Zhang N. Liu Y. X. Zhang X. Hu B. Qin Y.. (2018). Root microbiota shift in rice correlates with resident time in the field and developmental stage. Sci. China Life Sci. 61, 613–621. doi: 10.1007/s11427-018-9284-4 29582350

